# Long-Term Follow-up of the Woven EndoBridge (WEB) Device for the Treatment of Broad Based Intracranial Aneurysms: A Single-Center Retrospective Observational Analysis

**DOI:** 10.1007/s00062-025-01598-1

**Published:** 2025-12-05

**Authors:** Humberto Abraham Cortés Magdaleno, Ansgar Berlis, Guilherme Quint, Mahmoud Zaki, Christoph Maurer

**Affiliations:** 1 Department of Neuroradiology, University Hospital Augsburg, Augsburg, Germany; 2 Berufsgenossenschaftliche Unfallklinik Murnau, Murnau am Staffelsee, Germany

**Keywords:** Woven EndoBridge (WEB), Intracranial aneurysm, Endovascular treatment, Retreatment rate, Long-Term follow-up

## Abstract

**Purpose:**

Intracranial aneurysms are a major cause of hemorrhagic stroke, often requiring endovascular intervention. The Woven EndoBridge (WEB) device offers a minimally invasive solution for wide-neck bifurcation aneurysms and typically requires only single antiplatelet therapy, reducing bleeding risks associated with dual regimens. However, long-term clinical and angiographic data remain limited.

**Methods:**

This single-center, retrospective study analyzed 247 patients treated with the WEB device between January 2013 and December 2021, with clinical and imaging follow-up through June 2024. Demographics, aneurysm characteristics, procedural outcomes, and retreatment rates were evaluated. A competing risk model was applied to identify factors associated with retreatment.

**Results:**

The cohort included 247 patients (mean age: 63 years; 70% female) with 266 broad-based intracranial aneurysms. The overall retreatment rate was 12.8%, most occurring within the first year. Subgroup analysis revealed no significant differences based on aspect ratio (< 1.6 vs. ≥ 1.6) or device diameter (< 0.9 mm vs. ≥ 0.9 mm). Patients treated after 2018 had significantly lower retreatment rates (HR: 0.31; 95% CI: 0.13–0.71; *p* = 0.006), likely reflecting greater operator experience and introduction of the WEB17 model.

**Conclusion:**

The WEB device demonstrates durable aneurysm occlusion with a low long-term retreatment rate. Improved outcomes after 2018 suggest an effect of the learning curve and device refinement. Early imaging follow-up and continued operator training remain essential to optimize procedural success.

## Introduction

Intracranial aneurysms remain a leading cause of hemorrhagic stroke, necessitating innovative treatment approaches [1].

Since the International Subarachnoid Aneurysm Trial (ISAT), endovascular methods have emerged as the standard for managing ruptured aneurysms [2]. Techniques such as stent-assisted coiling, flow diverters, and neck-bridging devices have broadened the therapeutic options available [3].

The Woven EndoBridge (WEB) device (Terumo Neuro, Aliso Viejo, California, USA), introduced in 2010, provides a minimally invasive solution for wide-neck bifurcation aneurysms [4]. Unlike other devices that necessitate dual antiplatelet therapy, the WEB device typically requires only single antiplatelet therapy, reducing risk of secondary hemorrhage [5, 6]. While its safety and efficacy in acute settings have been demonstrated, long-term outcomes and retreatment data are less well understood [7].

Available evidence is often restricted to mid-term follow-up periods or selected patient populations, leaving key questions unanswered regarding the long-term angiographic stability and retreatment rates after WEB implantation. In particular, the influence of aneurysm morphology—such as aspect ratio—or device-related factors like sizing strategies remains controversial, with inconsistent findings across previous studies [8, 21, 22]. Despite recommendations for device oversizing to ensure stable deployment and complete neck coverage, its actual role in preventing retreatment has not been clearly defined.

Additionally, while early series have reported favorable occlusion rates [9], most studies have not systematically analyzed the effect of operator learning curves or the introduction of newer device models, such as the WEB17 system, on clinical outcomes. Given that device evolution and procedural experience may significantly influence retreatment risk, there is a need for real-world data assessing these parameters over extended follow-up periods.

Therefore, this study aims to evaluate long-term clinical, technical, and angiographic outcomes in patients treated with the WEB device at a tertiary care center, focusing on retreatment rates, morbidity, and predictive factors for aneurysm stability.

## Materials and Methods

This single-center, retrospective, observational study received approval from the local Institutional Review Board (ID 22-0219) and was conducted in accordance with the ethical standards of the Declaration of Helsinki (1964) and its subsequent revisions.

### Study Design

This retrospective, single-center study analyzed data from all patients treated with the Woven EndoBridge (WEB) device between January 2013 and December 2021 at a tertiary care neurointerventional center. Follow-up data were collected through June 2024 to provide a robust evaluation of long-term outcomes [7, 10].

### Study Population

The inclusion criteria comprised all patients treated for intracranial aneurysms with the WEB device at the participating center between January 2013 and March 2024. Patients were excluded from the study if relevant imaging or clinical follow-up information was incomplete or unavailable.

### Procedural Strategy

All interventions were carried out under general anesthesia using a biplane digital subtraction angiography system (Siemens, Erlangen, Germany). Patients undergoing treatment for unruptured aneurysms received dual antiplatelet therapy for at least five days prior to the procedure, which was continued postoperatively only when additional devices, such as stents, were used. For ruptured aneurysms, no antiplatelet therapy was administered prior to the intervention.

WEB device selection followed the manufacturer’s sizing guidance. After selection, the appropriate microcatheter (VIA 17, VIA 21, VIA 27, or VIA 33, Terumo Neuro, Aliso Viejo, California, USA) was advanced to the aneurysm under roadmap guidance using a working projection that avoided vessel overlap. The device was deployed with careful attention to maintaining catheter stability during detachment. A 10-minute observation period was generally observed prior to detachment. In selected cases, adjunctive techniques such as stent placement or coiling were employed based on individual anatomical or procedural requirements.

### Operator Experience

All procedures were performed by the same dedicated team of senior interventional neuroradiologists throughout the entire study period. No changes in the operating personnel occurred between 2013 and 2021. Each operator had more than ten years of experience in endovascular neurointerventions, ensuring a consistent level of technical expertise across all treatment years.

### Angiographic Follow-up and Occlusion Assessment

Angiographic occlusion was assessed on all follow-up imaging. In our center, follow-up is routinely performed using magnetic resonance angiography (MRA) or, when clinically indicated, digital subtraction angiography (DSA) at 6 months, followed by MRA at 18 months after the intervention. Occlusion status was graded using the Bicêtre Occlusion Scale Score (BOSS) [26], defined as:

- Grade 0: Complete occlusion
- Grade 0′: Complete occlusion with opacification of the proximal recess
- Grade 1: Opacification inside the WEB device
- Grade 2: Neck remnant
- Grade 3: Aneurysm remnant (contrast between the aneurysm wall and the WEB)
- Grade 1 + 3: Combined contrast inside and around the device

For analysis, Grades 0 and 0′ were classified as complete occlusion, whereas Grades 1, 2, 3, and 1 + 3 were collectively grouped as incomplete occlusion.

### Data Collection and Statistical Analysis

Clinical, procedural, and imaging data were reviewed to assess morbidity, procedural success, complications, aneurysm occlusion, and retreatment rates.

Descriptive statistics were used to summarize patient demographics, aneurysm characteristics, and procedural data, with continuous variables reported as means and standard deviations, and categorical variables as proportions.

Cumulative incidence plots were generated to illustrate the probability of retreatment over time, accounting for death as a competing event. Subgroup analyses were performed by stratifying patients based on the aspect ratio (< 1.6 vs. ≥ 1.6) and the ratio of aneurysm width to WEB device width (< 0.9 vs. ≥ 0.9). The cutoff of 0.9 was selected because it corresponds to the median value in the study population, allowing for a balanced comparison between smaller and larger ratios. Confidence intervals were included to represent the uncertainty associated with each estimate.

A competing risk regression model based on Fine and Gray’s method was applied to identify variables associated with the need for retreatment. Retreatment was defined as the primary event, and death was treated as a competing event. The variables assessed included age, aneurysm location, aneurysm angle, aspect ratio, maximum WEB device width and inflow angle. The inflow angle was defined as the angle between the parent vessel and the main axis of the aneurysm sac, reflecting the hemodynamic force direction entering the aneurysm. For interpretability of the hazard ratio (HR) estimates, the inflow angle was divided by 10, allowing the HR to reflect the risk of retreatment per 10-degree increment. Univariate models were used to select variables with a *p*-value < 0.2 for inclusion in the multivariable model. Baseline patient and aneurysm characteristics were additionally compared between aneurysms with and without retreatment using Welch’s t‑test for continuous variables and χ^2^ tests (or Fisher’s exact test where appropriate) for categorical variables. All analyses were performed on an aneurysm-based level to match the competing risk regression.

To evaluate the impact of operator experience, patients were divided into two groups based on the median treatment year (2018). A two-year follow-up censoring was applied to ensure consistent observation periods. All analyses considered a *p*-value ≤ 0.05 as indicative of statistical significance.

## Results

### Patients and Aneurysm Population

A total of 249 patients were treated with the WEB device between 2013 and 2021. Two patients were excluded from the final analysis due to insufficient imaging and clinical documentation, resulting in a study population of 247 patients (mean age: 63 years, 70% female) with 266 broad-based intracranial aneurysms (mean Aspect Ratio: 1.53). Follow-ups conducted through June 2024 assessed clinical and angiographic outcomes. Table 1 provides an overview of patient and aneurysm characteristics, including inflow angles, aspect ratios, and aneurysm-to-device width ratios. The median aneurysm width-to-WEB device width ratio was 0.9.

**Table 1:** Patient and Aneurysm Characteristics

Variable	*N* = 266^1^
*Age in years*	63 (13)
*Gender*	
Male	79 (30%)
Female	187 (70%)
*Location*	
Right	102 (38%)
Left	66 (25%)
Middle	98 (37%)
*ICA bifurcation*	11 (4.1%)
*ICA PCom*	19 (7.1%)
*Anterior choroidal artery*	1 (0.4%)
*AComA*	73 (27.4%)
*A2 segment ACA*	7 (2.6)
*Pericallosal artery*	2 (0.8%)
*M1 segment*	2 (0.8%)
*MCA bifurcation*	119 (44.7%)
*Vertebral artery*	2 (0.8%)
*PICA*	3 (1.1%)
*Basilar tip*	21 (7.9%)
*Superior cerebellar artery*	4 (1.5%)
*PCA P2 segment*	2 (0.8%)
*Inflow Angle*	144 (24)
Unknown	6
*Aneurysm Angle*	87 (16)
Unknown	5
*Aspect Ratio (SD)*	1.53 (0.54)
*Aspect Ratio*	
< 1.6	177 (67%)
> 1.6	89 (33%)
*Maximal Aneurysm width/Max WEB diameter*	0.91 (0.16)
Unknown	1
*Symptoms*	
Incidental	184 (69%)
SAH	63 (26%)
SAH + ICH	7 (2.6%)
ICH	5 (1.9%)
Recurrence after coiling	3 (1%)
Recurrence after clipping	2 (0.8%)
Recurrence after flow diverter	1 (0.4%)
Flow related AVM	1 (0.4%)

^1^Mean (SD); *n* (%)

Table 2 summarizes aneurysm characteristics before and after 2018. Patients treated after 2018 had significantly larger inflow angles (91°16 vs. 85°15, *p* = 0.003) and smaller maximum WEB diameters (0.89°0.14 vs. 0.93°0.17, *p* = 0.041), while aspect ratios remained comparable (*p* = 0.767).

**Table 2:** Aneurysm Characteristics—Subgroup Comparison

Characteristic	≤ 2018 (*N* = 175)	> 2018 (*N* = 91)	*p*
*Location*			0.099
Right	74 (42%)	28 (31%)	
Left	44 (25%)	22 (24%)	
Middle	57 (33%)	41 (45%)	
*Inflow Angle (Mean, SD)*	85 (15)	91 (16)	0.003 (*)
*Aspect Ratio (Mean, SD)*	1.53 (0.56)	1.51 (0.50)	0.767
*Max. Aneurysm width/Max. WEB Diameter (Mean, SD)*	0.93 (0.17)	0.89 (0.14)	0.041 (*)
*Aneurysm Size (Mean, SD)*	5.94 (2.65)	5.39 (2.26)	0.082

(*) indicates statistical significance (*p* < 0.05)

### Procedural Safety and Immediate Complications

Acute procedural outcomes were analysed for all 266 treated aneurysms. Intraprocedural rupture or perforation occurred in 7 cases (2.6%), typically related to mechanical micro-device manipulation or catheter–device friction. Acute thromboembolic or flow-related events were observed in 17 aneurysms (6.4%), necessitating rescue interventions such as antithrombotic medication, stent placement, or balloon assistance.

Technical issues involving device sizing or malposition required device retrieval or exchange in 12 procedures (4.5%). These included cases of WEB displacement outside the aneurysm sac and one instance of detachment failure requiring complete device removal and redeployment.

No aneurysm re-rupture after WEB implantation was documented. Overall mortality during follow-up was 4.9% (12/247 patients), with markedly higher rates in patients presenting with subarachnoid haemorrhage (SAH) (12.9%, 9/70) compared with unruptured aneurysms (1.7%, 3/177). In the SAH cohort, 8 of 9 deaths occurred within 30 days and were associated with high Hunt & Hess grades. In the unruptured cohort, no periprocedural deaths occurred; all three deaths occurred > 1 year post-procedure.

A total of six surviving patients (2.4%) experienced a long-term functional deficit (mRS > 2 at last follow-up) attributable to a procedural complication. Rescue strategies included stent, coil placement for perforation or neck remnant sealing, balloon-assisted techniques, and pharmacologic agents such as tirofiban (Aggrastat) or papaverine.

### Angiographic Occlusion Results

Angiographic occlusion was evaluated at all follow-up intervals using the Bicêtre Occlusion Scale Score (BOSS) [26]. Immediate postprocedural imaging (*N* = 266 aneurysms) demonstrated complete occlusion (BOSS 0/0′) in 62.0%. At 1‑year follow-up (*N* = 188), complete occlusion was observed in 67.0%, increasing to 75.6% at 3 years (*N* = 119). Residual neck remnants (BOSS 2) decreased from 12.8% at 1 year to 10.1% at 3 years, indicating progressive thrombosis over time.

A dedicated long-term analysis was performed in the subgroup with ≥ 5 years of clinical follow-up (*N* = 66). In this cohort, 12 patients (18.2%) underwent retreatment. Among the remaining 54 clinically stable patients (81.8%), BOSS data were available for 52 aneurysms. Of these, 84.6% demonstrated durable complete occlusion (BOSS 0/0′), and 15.4% exhibited minor residuals (BOSS 1 or BOSS 2). Notably, three retreatments (25.0%) in this subgroup occurred more than five years after the index procedure, underscoring the need for continued long-term surveillance.

### Treatment Results

The overall retreatment rate was 12.8%, with the majority occurring within the first year post-procedure. Among the 128 patients treated before, 2018, 27 (21.1%) required retreatment, compared to only 10 out 138 patients (7.2%) treated in or after 2018. Figure 3 details retreatment incidence stratified by aneurysm characteristics and treatment periods. Patients treated after 2018 had significantly lower retreatment rates (HR: 0.31; 95% CI: 0.13–0.71; *p* = 0.006).

### Baseline Comparison Between Retreated and Non-Retreated Aneurysms

When comparing baseline characteristics between aneurysms with and without retreatment (Table 3), age, aneurysm angle, aspect ratio, aneurysm-to-device width ratio, aneurysm location, presenting symptoms, and treatment period did not differ significantly between groups (all *p* > 0.05). In contrast, aneurysms that required retreatment had a significantly higher inflow angle (152.7° ± 19.1° vs. 142.3° ± 24.4°, *p* = 0.006) and were treated with larger WEB devices (7.9 ± 2.0 mm vs. 5.6 ± 1.9 mm, *p* < 0.001). The proportion of female patients was lower in the retreatment group compared with the non-retreatment group (50.0% vs. 73.3%, *p* = 0.010).

**Table 3:** Baseline characteristics of aneurysms with and without retreatment

	No Retreatment	Retreatment	*p*-value
*Continuous Variables*			
Age, years	63.3 ± 13.3 (*n* = 232)	63.4 ± 14.1 (*n* = 34)	0.947
Inflow angle	142.3 ± 24.4 (*n* = 226)	152.7 ± 19.1 (*n* = 34)	0.006
Aneurysm angle	87.3 ± 16.0 (*n* = 227)	85.5 ± 13.3 (*n* = 34)	0.474
Aspect ratio	1.51 ± 0.51 (*n* = 232)	1.60 ± 0.73 (*n* = 34)	0.501
Aneurysm width/WEB width ratio	0.91 ± 0.17 (*n* = 231)	0.94 ± 0.15 (*n* = 34)	0.281
WEB diameter, mm*	5.61 ± 1.89 (*n* = 231)	7.92 ± 1.99 (*n* = 34)	< 0.001*
*Sex*			
Female	170 (73.3%)	17 (50.0%)	0.010*
Male	62 (26.7%)	17 (50.0%)	
*Aspect Ratio Category*			
< 1.6	153 (65.9%)	24 (70.6%)	0.733
≥ 1.6	79 (34.1%)	10 (29.4%)	
*Presenting Symptoms*			
SAH	52 (26.1%)	6 (22.2%)	0.873
Incidental	133 (66.8%)	21 (77.8%)	
Recurrence after clipping	2 (1.0%)	0 (0%)	
Recurrence after coiling	2 (1.0%)	0 (0%)	
Flow-related AVM	1 (0.5%)	0 (0%)	
SAH + ICH	6 (3.0%)	0 (0%)	
ICH	3 (1.5%)	0 (0%)	

Mean (SD) for continuous variables; *n* (%)* for categorical variables

*p*-values from Welch’s t‑test (continuous) or Chi-square test (categorical)

**Table 4:** Hazard Ratios for Retreatment Risk Factors. This table presents the results of the competing risk regression analysis evaluating factors associated with the risk of retreatment. In the univariable analysis, inflow angle (per 10° increment) was a statistically significant predictor of retreatment, while other morphological parameters were not.

Variable	HR (95% CI) Univariable	*p*-value Univariable	HR (95% CI) Multivariable	*p*-value Multivariable
Age	1.01 (0.98, 1.05)	0.36	–	–
Location (L vs. R)	0.93 (0.36, 2.40)	0.88	–	–
Location (L vs. M)	1.46 (0.68, 3.11)	0.33	–	–
Inflow Angle (per 10°)	1.14 (1.00, 1.29)	0.046	1.12 (0.99, 1.27)	0.076
Aneurysm Angle	0.99 (0.97, 1.01)	0.40	–	–
Aspect Ratio	1.11 (0.60, 2.05)	0.73	–	–
Max. Aneurysm width/Max. WEB Diameter	6.01 (0.58, 62.7)	0.13	4.69 (0.43, 50.8)	0.20

### Subgroup Analysis

**Fig. 1:**
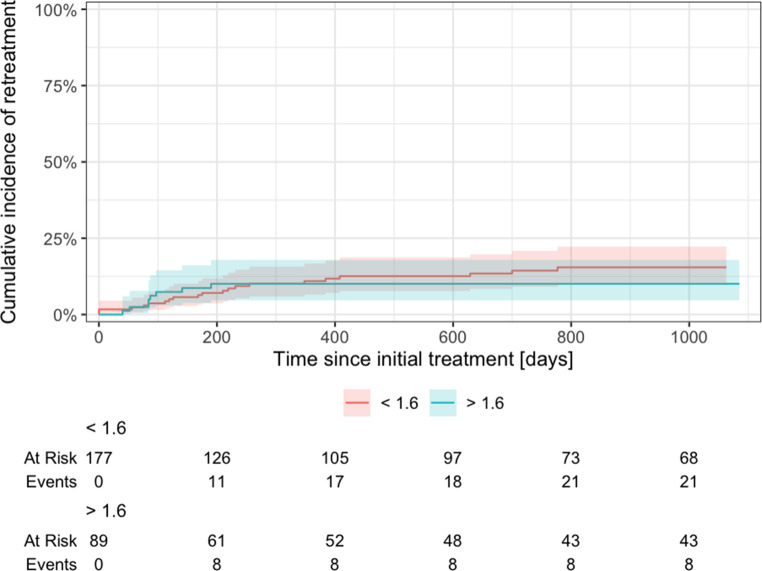
Cumulative incidence of retreatment after WEB implantation using a competing-risk framework (death as competing event). Curves are stratified by aspect ratio (AR) < 1.6 vs. ≥ 1.6; shaded areas indicate 95% confidence intervals (*CIs*). (*AR* aspect ratio, *CI* confidence interval, *WEB* Woven EndoBridge)

**Fig. 2:**
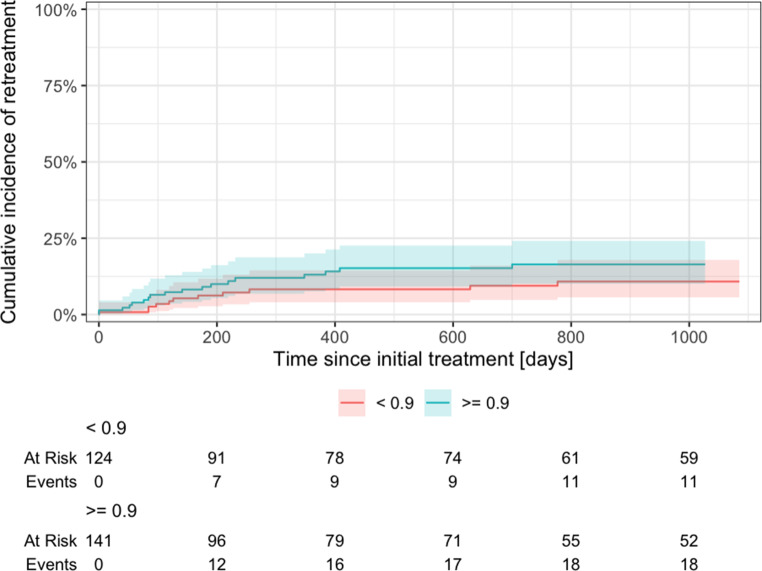
Cumulative incidence of retreatment stratified by aneurysm-to-WEB device width ratio < 0.9 vs. ≥ 0.9. Curves were generated using a competing-risk model as in Fig. 1; 95% CIs are displayed. (*CI* confidence interval, *WEB* Woven EndoBridge)

**Fig. 3:**
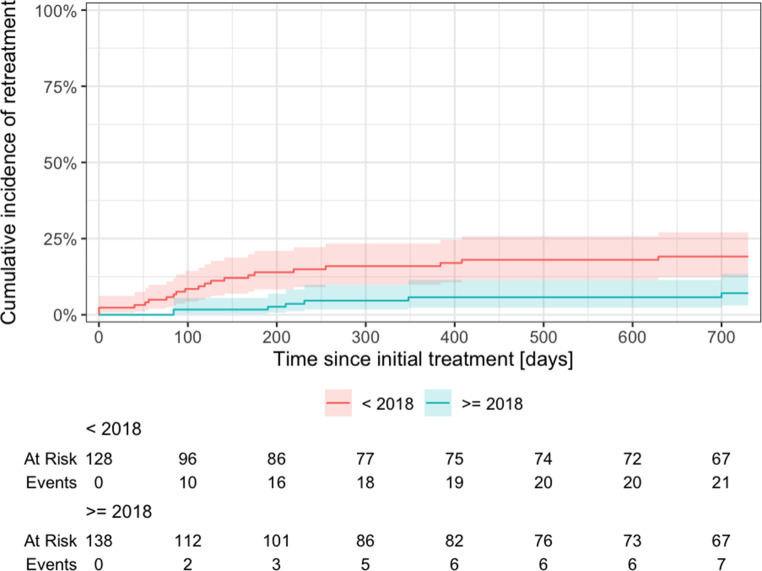
Cumulative incidence of retreatment by treatment period (< 2018 vs. ≥ 2018), applying two-year follow-up censoring to ensure comparable observation windows. Shaded areas show 95% CIs. This analysis reflects the potential learning-curve effect and introduction of the WEB 17 device. (*CI* confidence interval, *WEB* Woven EndoBridge)

**Fig. 4:**
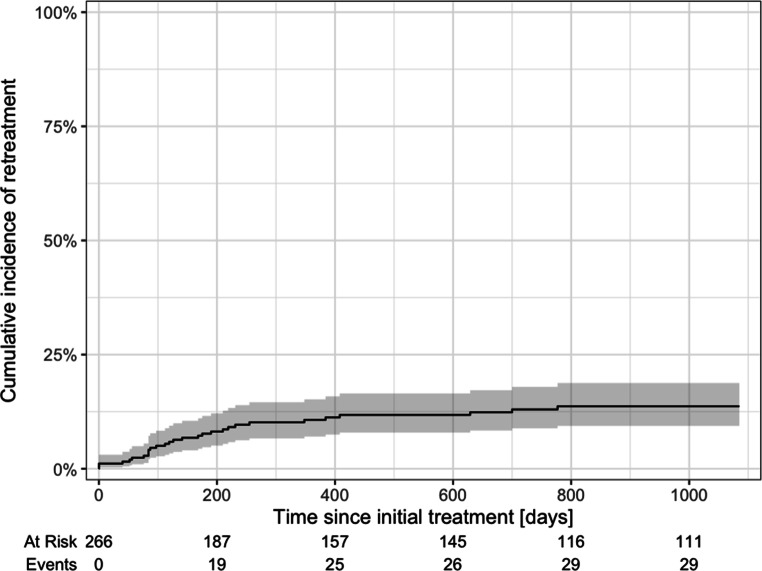
Cumulative incidence of retreatment according to inflow-angle categories (grouped in 10-degree increments; see *Materials and Methods* for definition). Shaded bands represent 95% CIs. (*CI* confidence interval, *WEB* Woven EndoBridge)

Cumulative incidence plots (Figs. 1, 2, 3 and 4) illustrate retreatment rates stratified by aspect ratio, aneurysm-to-device width ratio, and treatment period.

#### Aspect Ratio

The overlapping confidence intervals and the crossing of curves at the beginning suggest no significant differences in retreatment rates between the groups over time.

#### Aneurysm-to-Device Width Ratio

The WEB device was typically oversized relative to the aneurysm width to ensure stable deployment and full neck coverage, with the literature recommending an oversizing of 1 mm to 2 mm beyond the maximum aneurysm width [7]. This approach aims to maximize aneurysm occlusion and minimize the risk of migration or incomplete sealing. Despite this standardized oversizing strategy, our findings suggest that the aneurysm-to-device width ratio was a mild but statistically significant predictor of retreatment risk (*p* = 0.041).

### Five-Year Subgroup Analysis

Among the 66 patients with at least five years of follow-up, 12 (18.2%) required retreatment, while 54 (81.8%) remained stable without further intervention. The median time to retreatment was 319 days after the initial WEB implantation. Notably, 3 of 12 retreatments (25%) occurred more than five years after the initial procedure, underscoring the need for sustained long-term surveillance in selected cases.

#### Treatment Period

The median procedure year (2018) was selected as it represents the midpoint of the study period (2013–2021). To ensure comparability between groups, follow-up was limited to two years. As illustrated in Fig. 3, patients treated after 2018 had a significantly lower retreatment risk (HR: 0.31, 95% CI: 0.13–0.71; *p* = 0.006).

The majority of retreatments occurred within the first year after treatment, with differences observed across subgroups.

#### Inflow Angle

In the competing risk regression analysis, the inflow angle (scaled per 10-degree increment) was associated with an increased risk of retreatment in the univariable model (HR: 1.14; 95% CI: 1.00–1.29; *p* = 0.046). After adjustment for other variables in the multivariable model, this association showed a trend toward significance but did not meet the conventional threshold (HR: 1.12; 95% CI: 0.99–1.27; *p* = 0.076).

## Discussion

Our study demonstrates the consistent efficacy of the WEB device in treating wide-neck bifurcation aneurysms, as evidenced by our cohort’s low retreatment rate of 12.8% over a follow-up period extending to 2024. This aligns with previous studies highlighting the device’s utility in achieving durable aneurysm occlusion while minimizing procedural complexity. For example, a recent meta-analysis of 29 studies (*n* ≈ 2067) found an overall WEB retreatment rate of ~8.6% (95% CI: 6.5–10.9) after initial treatment with the device [23]. Similarly, the combined WEBCAST/French Observatory/WEBCAST-2 European studies reported a retreatment rate of 9.3% at two-year follow-up [24]. Other series have reported retreatment rates ranging from ~5–7% in mid-term follow-up cohorts, underlining the variability in outcomes depending on aneurysm morphology and device sizing [25]. These findings reinforce the notion that the WEB device is a reliable alternative to traditional techniques such as stent-assisted coiling or flow diversion, particularly for complex aneurysms requiring specialized approaches.

### Long-Term Efficacy of the WEB Device

The cumulative incidence of retreatment in our study was highest within the first year after the procedure, aligning with previously published data. For instance, Peterson et al. (2015) reported a 10% retreatment rate at 1‑year follow-up in their multicenter series of aneurysms treated with the WEB device, noting that most retreatments occurred during the early follow-up period [12]. Similarly, Caroff et al. (2023) observed that the majority of aneurysm recurrences or residual neck issues necessitating further intervention were detected within the first year, resulting in a retreatment rate close to 10% by 12 months [13]. These findings corroborate our observed retreatment rate, emphasizing the importance of vigilant imaging follow-up and clinical assessment during the initial postprocedural year. Despite this early vulnerability, the retreatment rates remained relatively low, emphasizing the device’s durability when properly deployed.

In a dedicated subgroup analysis of 66 patients with a follow-up duration of at least five years, 12 individuals (18.2%) underwent retreatment, while 54 (81.8%) remained stable without the need for further intervention. The median time to retreatment in this group was 319 days after the initial WEB implantation, demonstrating that most reinterventions in this subgroup still occurred within the first year. However, 3 of these 12 retreatments (25%) were performed more than five years after the initial treatment. These findings highlight the durable performance of the WEB device in the majority of patients, while also indicating that very late retreatments, although infrequent, can still arise and should be accounted for in long-term follow-up strategies.

### Subgroup Analyses: Aspect Ratio and Device Size

One of the central aims of this study was to explore factors influencing the need for retreatment. Our subgroup analyses revealed no significant differences in retreatment rates based on aneurysm aspect ratio (< 1.6 vs. ≥ 1.6) or WEB device diameter (< 0.9 mm vs. ≥ 0.9 mm). These findings are particularly noteworthy as they challenge the conventional understanding that aneurysm morphology, such as aspect ratio, plays a decisive role in procedural success [9]. Instead, our results suggest that deployment technique and device stability may be more critical determinants of long-term outcomes, an observation consistent with reports emphasizing operator expertise, precise sizing, and vigilant follow-up to detect early recurrences [5, 6, 12–14]. Although our confidence intervals were broad due to the limited number of retreatment events, the lack of significant morphological effects implies that the WEB device performs reliably across a spectrum of aneurysm shapes. This versatility broadens the indications for the WEB system, underscoring its potential utility in cases previously deemed unsuitable for intrasaccular flow disruption.

In addition to aspect ratio, we also examined the impact of sizing strategy on retreatment risk. The WEB device was typically oversized relative to the aneurysm width to ensure stable deployment and full neck coverage, with the literature recommending an oversizing of 1–2 mm beyond the maximum aneurysm width [7]. This approach is designed to maximize aneurysm occlusion and minimize the risk of migration or incomplete sealing. Despite this standardized sizing strategy, our analysis revealed that the aneurysm-to-device width ratio was a mild but statistically significant predictor of retreatment risk (*p* = 0.041). This finding suggests that even small deviations in sizing precision may affect long-term treatment durability, emphasizing the importance of individualized device selection and meticulous procedural planning.

### Learning Curve and Temporal Trends

A significant finding in our study was the marked improvement in outcomes for patients treated in or after 2018. Retreatment risk was significantly lower in this group (HR: 0.31; 95% CI: 0.13–0.71; *p* = 0.006), suggesting a learning curve effect.

Additionally, this improvement coincides with the introduction of the WEB 17 device, which features a smaller profile and enhanced deliverability compared with its predecessor, WEB 21. The WEB 21 system typically requires a 0.021-inch microcatheter and is available in standard diameter increments ranging from 4 to 7 mm. In contrast, the newer WEB 17 system is compatible with a smaller 0.017-inch microcatheter and offers a broader spectrum of sizing options starting from 3 mm, allowing for more tailored device selection in smaller or distally located aneurysms [16]. These technical refinements allow safer navigation in tortuous vessels and potentially reduce the risk of procedural complications. König et al. similarly noted the expanding indications of WEB 17 toward smaller and more distally located aneurysms, reflecting a trend toward improved outcomes and fewer retreatments [14]. This trend is further supported by findings from the CLEVER study, a prospective multicenter evaluation of the WEB 17 system, which reported an adequate occlusion rate of 82.2% and a notably low retreatment rate of 2.6% at 12-month follow-up in both ruptured and unruptured aneurysms [20]. These world data provide strong evidence for the effectiveness of the WEB 17 device and reinforces the notion that both device refinement and procedural experience contribute meaningfully to improved clinical outcomes.

The learning curve effect has been well-documented in neurointerventional procedures, where operator familiarity with device-specific nuances, such as sizing and deployment, significantly influences success rates [11]. In our study, the consistent use of an “oversizing” strategy for the WEB device, informed by growing experience and supported by 3D imaging tools, likely contributed to the observed improvement in outcomes. These findings emphasize the importance of ongoing training and knowledge sharing within the neurointerventional community to ensure that operators are equipped to maximize the device’s potential.

### Procedural Safety and Antiplatelet Therapy

Safety remains a cornerstone of any endovascular treatment, and our findings reaffirm the WEB device’s favorable safety profile. The use of single antiplatelet therapy in most cases mitigates bleeding risks, making the WEB device particularly suitable for older patients or those with contraindications to dual antiplatelet therapy. Despite the overall safety, the choice and duration of antiplatelet therapy remain subjects of debate. For example, Goyal et al. reported in their ‘How to WEB’ methodology that no dual antiplatelet therapy (DAPT) is required for ruptured aneurysms as long as the WEB device remains fully contained within the aneurysm dome, thereby minimizing thrombotic risk [7]. Conversely, for unruptured aneurysms, pretreatment with DAPT is often recommended to facilitate possible adjunctive stenting and reduce intraprocedural thromboembolism. After successful device deployment and confirmation that the WEB device is fully contained, many operators taper to single-agent aspirin, reserving longer or dual regimens for borderline device protrusion or complex anatomy. Ultimately, this variability underscores the absence of a universal post-WEB antiplatelet protocol, emphasizing the importance of tailoring treatment to specific aneurysm characteristics, procedural nuances, and patient risk profiles.

### Inflow Angle

Another factor that may influence retreatment risk is the inflow angle, which reflects the angulation between the parent artery and the aneurysm axis. In our cohort, a higher inflow angle was associated with an increased likelihood of retreatment in the univariable competing risk regression model (HR: 1.14 per 10° increment; 95% CI: 1.00–1.29; *p* = 0.046). Although the association lost statistical significance in the multivariable model (HR: 1.12; 95% CI: 0.99–1.27; *p* = 0.076), the trend suggests that steep inflow angles may contribute to suboptimal flow disruption or incomplete aneurysm occlusion. This is consistent with previous hemodynamic analyses showing that larger inflow angles are associated with higher peak flow velocity, increased wall shear stress, and more complex flow patterns within the aneurysm sac, and WEB shape modification, potentially leading to less stable occlusion and higher retreatment rates [17, 18]. Notably, beginning in 2018, patient selection in our center became more conservative regarding inflow angles, with steeper configurations often being excluded from WEB treatment. This shift in clinical practice may have contributed to the observed decrease in retreatment rates among patients treated after 2018, as illustrated in Fig. 3. While our data supports a potential role of inflow angle as a predictor of retreatment, the limited number of events in this study restricts definitive conclusions. Larger cohorts with detailed morphological and hemodynamic assessments are needed to further explore this association and its implications for patient selection or device sizing.

### Implications for Clinical Practice

The consistent performance of the WEB device across various aneurysm morphologies and its adaptability to different clinical scenarios underscore its value as a versatile tool in neurointervention. The observed learning curve effect highlights the need for structured training programs and procedural standardization to ensure consistent outcomes across operators and centers. Additionally, the lack of significant predictors of retreatment in our multivariable analysis suggests that clinicians should adopt a comprehensive approach to patient selection and procedural planning, rather than relying solely on morphological parameters.

### Limitations and Future Directions

While our study provides insights into the long-term outcomes of the WEB device, several limitations must be acknowledged. The retrospective, single-center design introduces potential selection bias, and the small number of retreatment events limits the statistical power of subgroup analyses. Future multicenter studies with larger cohorts are needed to validate our findings and explore additional predictors of procedural success.

Furthermore, as newer iterations of the WEB device are introduced, such as those designed for even smaller aneurysms or off-label applications, it will be essential to evaluate their impact on long-term outcomes. Prospective registries and randomized controlled trials could provide the high-quality evidence needed to guide clinical decision-making and optimize patient care.

## Conclusion

This study demonstrates the long-term efficacy and safety of the WEB device for treating wide-neck bifurcation aneurysms, with most retreatments occurring within the first year, highlighting the importance of early follow-up. Improved outcomes after 2018 suggest a learning curve effect probably related to operator experience advancements in device design and stricter consideration of aneurysm morphology. While the WEB device remains a minimally invasive option, further multicenter studies are needed to validate these findings and confirm predictive factors for retreatment.
