# Correction to: Long-Term Follow-up of the Woven EndoBridge (WEB) Device for the Treatment of Broad Based Intracranial Aneurysms: A Single-Center Retrospective Observational Analysis

**DOI:** 10.1007/s00062-026-01647-3

**Published:** 2026-03-30

**Authors:** Humberto Abraham Cortés Magdaleno, Ansgar Berlis, Guilherme Quint, Mahmoud Zaki, Christoph Maurer

**Affiliations:** 1 Department of Neuroradiology, University Hospital Augsburg, Augsburg, Germany; 2 Berufsgenossenschaftliche Unfallklinik Murnau, Murnau am Staffelsee, Germany

**Correction to:**

**Clin Neuroradiol 2025**

10.1007/s00062-025-01598-1

The publication of this article unfortunately contained mistakes. The order of the authors was not correct. The corrected order is given below.

- Wrong order:

Humberto Abraham Cortés Magdaleno · Christoph Maurer · Ansgar Berlis · Guilherme Quint · Mahmoud Zaki

- Correct order:

Humberto Abraham Cortés Magdaleno · Ansgar Berlis · Guilherme Quint · Mahmoud Zaki · Christoph Maurer

The original article has been corrected.

